# Circumvention of ara-C resistance by aphidicolin in blast cells from patients with AML

**DOI:** 10.1054/bjoc.2000.1639

**Published:** 2001-03

**Authors:** J M Sargent, A W Elgie, C J Williamson, G M Lewandowicz, C G Taylor

**Affiliations:** Haematology Research, Pembury Hospital, Pembury, Kent, TN2 4QJ, UK

**Keywords:** aphidicolin, DNA repair, drug resistance, AML, ara-C

## Abstract

Treatment failure in AML is often attributed to P-glycoprotein-associated multidrug resistance. However, the importance of increased DNA repair in resistant cells is becoming more apparent. In order to investigate the ability of the DNA repair inhibitor aphidicolin to modulate drug resistance, we continually exposed blasts cells, isolated from 22 patients with AML, to a variety of agents ± 15 μM aphidicolin for 48 hours. Cell survival was measured using the MTT assay. Overall, there was no significant effect of aphidicolin on sensitivity to daunorubicin, doxorubicin, etoposide or fludarabine. However, there was a marked increase in sensitivity to ara-C with a median 4.75-fold increase overall (range 0.8–80-fold;*P*< 0.005). The effect of aphidicolin was significantly greater in blast cells found resistant in vitro to ara-C (8.9-fold compared to 2.12-fold, *P*< 0.01). This observation was further validated by the correlation between ara-C LC _50_ and extent of modulation effect (*P*< 0.05). Cells isolated from 10 cord blood samples were also tested in order to establish the haematological toxicity of combining ara-C and aphidicolin. The therapeutic index (LC _50_ normal cells/tumour cells) for ara-C + aphidicolin was higher than that for ara-C alone suggesting no increased myelotoxicity for the combination. Increased cytotoxicity without increased haematotoxicity makes the combination of ara-C plus aphidicolin ideal for inclusion in future clinical trials. © 2001 Cancer Research Campaign http://www.bjcancer.com


*British Journal of Cancer* (2001) **84**(5), 680–685
© 2001 Cancer Research Campaign
doi: 10.1054/ bjoc.2000.1639, available online at http://www.idealibrary.com on IDEAL

http://www.bjcancer.com

### Circumvention of ara-C resistance by aphidicolin in blast cells from patients with AML

**JM Sargent, AW Elgie, CJ Williamson, GM Lewandowicz and CG Taylor**

Haematology Research, Pembury Hospital, Pembury, Kent TN2 4QJ, UK

**Summary** Treatment failure in AML is often attributed to P-glycoprotein-associated multidrug resistance. However, the importance of increased DNA repair in resistant cells is becoming more apparent. In order to investigate the ability of the DNA repair inhibitor aphidicolin to modulate drug resistance, we continually exposed blasts cells, isolated from 22 patients with AML, to a variety of agents ± 15 μM aphidicolin for 48 hours. Cell survival was measured using the MTT assay. Overall, there was no significant effect of aphidicolin on sensitivity to daunorubicin, doxorubicin, etoposide or fludarabine. However, there was a marked increase in sensitivity to ara-C with a median 4.75-fold increase overall (range 0.8–80-fold; $P < 0.005$). The effect of aphidicolin was significantly greater in blast cells found resistant in vitro to ara-C (8.9-fold compared to 2.12-fold, $P < 0.01$). This observation was further validated by the correlation between ara-C $LC_{50}$ and extent of modulation effect ($P < 0.05$). Cells isolated from 10 cord blood samples were also tested in order to establish the haematological toxicity of combining ara-C and aphidicolin. The therapeutic index ($LC_{50}$ normal cells/tumour cells) for ara-C + aphidicolin was higher than that for ara-C alone suggesting no increased myelotoxicity for the combination. Increased cytotoxicity without increased haematotoxicity makes the combination of ara-C plus aphidicolin ideal for inclusion in future clinical trials. © 2001 Cancer Research Campaign http://www.bjcancer.com

**Keywords**: aphidicolin; DNA repair; drug resistance; AML; ara-C

Whilst prognosis has improved dramatically over recent years for some childhood leukaemias, this is not the case for adult acute myeloid leukaemia (AML). Patients with AML are treated with a combination regimen containing an anthracycline teamed with cytosine arabinoside (ara-C). Initial remission rates are typically around 70% (Åström et al, 2000), however patients tend to relapse early and less than 25% can expect to have a sustained, long-term survival. Drug resistance remains one of the major factors leading to treatment failure. Multidrug resistance (MDR) associated with overexpression of P-glycoprotein is prevalent in haematological malignancies. Whilst some MDR modulation regimens have shown promise, generally early results have been disappointing (Covelli, 1999).

Interestingly, whilst the in vitro anthracycline sensitivity of blast cells from patients with AML predicts initial response to therapy (Sargent et al, 1997), a recent study suggests that it is in vitro resistance to the antimetabolite ara-C that correlates with early relapse (Klumper et al, 1996). Resistance to ara-C can be attributed to many factors but alterations in phosphorylation of ara-C to its active metabolite ara-CTP has been widely believed to be the rate-limiting step leading to the incorporation of ara-CTP into DNA with subsequent induction of DNA strand breaks (Freund et al, 1998). This is thought to cause termination of chain elongation and switch on apoptosis by a mechanism not yet fully understood. Circumvention of resistance to ara-C has therefore centred on compounds which can increase conversion to the active metabolite by directly increasing cellular phosphorylation e.g. bryostatin (Elgie et al, 1998) or fludarabine by increasing deoxycitidine kinase activity (Plunkett and Gandhi, 1993).

A novel approach to the problem of drug resistance in AML would be to exploit the fact that drug-resistant cells often have an increased capacity to repair DNA damage caused by cytotoxic attack (Barret and Hill, 1998). By attacking a resistance mechanism common to many different classes of cytotoxic agent rather than using resistance modulators to intervene in specific pathways, we hoped to address the issue of pleiotropic drug resistance. We have previously shown that it is possible to inhibit repair of DNA/platinum adducts using the DNA polymerase inhibitor aphidicolin in cells from patients with ovarian cancer (Sargent et al, 1996). Moreover, we found that aphidicolin had a greater effect in cells found resistant in vitro to the platinum agents.

Aphidicolin glycinate, the water-soluble analogue of aphidicolin entered Phase I trials and results showed that whilst toxicity was limited, there did not appear to be significant antitumour effect when given as a single agent (Sessa et al, 1991). Preclinical studies suggested this compound may be effective when combined with a platinum agent but the clinical studies were not completed and the true worth of this compound has never been determined.

In this report, we have investigated the use of aphidicolin to modulate resistance to the DNA damaging agents commonly used to treat AML. Results of a pilot study have been published in extended abstract form (Sargent et al, 1999). As the major toxicity of ara-C is associated with myelosuppression, we also investigated the haematological toxicity of its combination with aphidicolin using stem cell rich cord blood samples.

*Received 24 August 2000*
*Revised 20 November 2000*
*Accepted 23 November 2000*

*Correspondence to:* J Sargent

### MATERIALS AND METHODS

### Patients and samples

23 samples were received from 22 patients with AML; 19 with de novo AML (13 on first presentation, 5 on relapse and 1 patient tested twice – on presentation and relapse) and 3 with AML 2˚ to MDS all of whom were chemotherapy naive. Approximately 5 ml of bone marrow (10 samples) or 40 ml of peripheral blood (13 samples) were collected into citrate phosphate dextrose and tested within 48 hours. Blast cells were harvested using density gradient centrifugation (Histopaque, Sigma) and washed twice in RPMI 1640. A final cell suspension was prepared at approximately $1–2 \times 10^6$ blasts $ml^{-1}$ RPMI 1640 plus 10% fetal calf serum (FCS), 100 IU $ml^{-1}$ penicillin, 100 µg $ml^{-1}$ streptomycin (all Sigma, Poole, UK). Morphology was checked using May Grünwald Giemsa stain on a cytospin of the final cell preparation. All samples contained >80% blast cells.

### Drug exposure

Blast cells were continuously exposed at 37˚C, 5% $CO_2$ for 48 hours in 96-well microtitre plates to 4 concentrations of cytotoxic drugs commonly used to treat AML. Drugs tested included the antimetabolites: ara-C, fludarabine (Flud); anthracyclines: daunorubicin (DNR), doxorubicin (DOX); epipodophyllotoxin: etoposide (Etop). Aphidicolin (Sigma, Poole, UK) was tested as a single agent in 12 cases and did not appear to be cytotoxic up to a concentration of 30 µM. Therefore, continuous exposure to cytotoxic agents took place in the presence or absence of a fixed concentration of aphidicolin (15 µM) the concentration found to inhibit DNA repair in our previous study (Sargent et al, 1996). Control wells contained cells plus medium or, to control the co-incubation experiments, medium plus aphidicolin at 15 µM.

### MTT assay

The method used was similar to that previously described (Sargent and Taylor, 1989). After 48 hours the drugs and medium were removed from the wells by flicking and 50 µl of MTT solution (2 mg MTT $ml^{-1}$ Hanks balanced salt solution without phenol red) was added to all wells. The microtitre plates were re-incubated for 4 hours and any formazan crystals formed were dissolved in acid/alcohol (0.04 N HCl in isopropanol). The plates were read at 570 nm (reference 690 nm) on an Anthos 2001 plate reader.

The $LC_{50}$ (drug concentration lethal for 50% of cells) was calculated or predicted using our own customized software for each experiment. Patients were deemed sensitive if the $LC_{50}$ value for individual drugs fell below the cut off points given in Table 1, resistant if above. These cut-off values have been previously validated by correlation with the induction of remission (Sargent and Taylor, 1989; Sargent et al, 1997)

The fold difference in cytotoxicity on co-incubation with aphidicolin was measured as the sensitization ratio: $LC_{50}$ of drug over $LC_{50}$ in the presence of aphidicolin therefore, positive modulation effects were indicated by a ratio >1.0.

### DNA analysis

Apoptotic cells, analysed using flow cytometry, are clearly distinguishable as distinct subpopulations using DNA content and light scatter measurements (Sherwood and Schimke, 1995). Cells were exposed to ara-C ± aphidicolin for 48 hours then pelleted and fixed by dropwise addition of 70% ice-cold ethanol while vortexing. Controls consisted of blast cells incubated in medium or medium + aphidicolin at 15 µM for 48 hours. Cells were then stored at 4˚C for up to 1 month until required. After the addition of RNase (1 mg $ml^{-1}$; Sigma, Poole), cells were stained with propidium iodide (PI; 400 mg $ml^{-1}$ PBS; Sigma, Poole) for 30 min at 37˚C. A Coulter Epics XL flow cytometer with an argon laser tuned to 488 nm was used to analyse the DNA content of the cells by measuring forward and orthogonal light scatter and red fluorescence. A plot of side scatter against PI staining was used to gate the sub $G_1$ population of cells to give the percentage of cells with reduced DNA content. When these cells were examined under a fluorescent microscope, they showed the typical signs of apoptosis with chromatin condensation, cell shrinkage and blebbing of the membrane. The percentage of remaining viable cells was calculated.

### Cord blood samples

In order to establish the haematological toxicity of ara-C and aphidicolin in combination, cells were obtained from cord blood samples from 10 mothers, after informed consent. These cells were prepared in the same way as the cells from AML patients, then frozen in liquid nitrogen in RPMI 1640 with 20% FCS and 10% DMSO until required. After defrosting, the cells were washed in medium to remove the DMSO then re-suspended at $1 \times 10^6$ $ml^{-1}$

**Table 1** Chemosensitivity and modulation by aphidicolin

| Drug | $LC_{50}$ median (range; µM) | $LC_{50}$ + aph median (range; µM) | Sensitization ratio median (range) | Number SR>2/number tested | Cut-off for resistance (µM) | Resistance rate (%) |
|---|---|---|---|---|---|---|
| ara-C | 5.02 (0.25–41) | 0.91* (0.08–41) | 4.75 (0.8–80) | 16/23 | 2.5 | 15 (65) |
| Flud | 3.09 (0.9–24) | 5.01 (1.5–70) | 0.66 (0.34–2.05) | 1/7 | 20 | 2 (29) |
| DNR | 0.8 (0.07–3.9) | 0.53 (0.04–2.84) | 1.38 (0.71–4.15) | 1/11 | 1.0 | 1 (9) |
| DOX | 0.83 (0.16–2.07) | 0.82 (0.02–2.07) | 1.07 (0.71–1.75) | 1/6 | 1.0 | 2 (33) |
| Etop | 44.2 (4.25–93) | 41.5 (5.95–77) | 1.11 (0.71–1.75) | 0/13 | 25 | 10 (77) |

* $P$ = < 0.005.

© 2001 Cancer Research Campaign 

in RPMI 1640 with 10% FCS and antibiotics. Cells were exposed to ara-C ± aphidicolin as above. This was followed by the MTT assay and the $LC_{50}$ value was calculated for each sample. A $CD34^+$ haematopoietic stem cell count was performed according to the published guidelines (Barnett et al, 1999). The CD34-PE and CD45-FITC antibodies (Beckman Coulter, UK) were used and the samples read on a Coulter Epics flow cytometer using the ISHAGE sequential gating strategy.

### Statistics

Non-parametric methods were used throughout. Wilcoxon signed rank test was used to assess the effect of aphidicolin, with paired data. The Mann Whitney *U* test was used to assess differences between groups of patients. The Spearman's rank correlation coefficient was used to compare drug $LC_{50}$ values with sensitization ratios. A $P$ value of $\leq 0.05$ was considered significant.

### RESULTS

### Drug sensitivity

There was a marked variation in the effect of the antimetabolites and etoposide between patients (Table 1). This variation was not so pronounced for the anthracyclines, with a low overall resistance rate in this group of patients.

### Modulation by aphidicolin

Table 1 shows the median and range of $LC_{50}$ values obtained for each drug ± aphidicolin. The greatest effect overall was seen for ara-C ($P < 0.005$). Up to 80-fold increases in sensitivity were seen for 21 of the 23 samples tested, 16 (70%) having >2.0-fold augmentation. Interestingly, aphidicolin had no significant effect on the sensitivity of the other antimetabolite, fludarabine and had minimal effect on the cytotoxicity of the anthracyclines or etoposide in this data set. There did not appear to be any correlation of this effect with previous treatment or whether AML was the primary disease or secondary to MDS.

### Relationship between sensitivity to ara-C and modulation by aphidicolin

There was a significant correlation between the $LC_{50}$ values for ara-C and the sensitization ratio calculated from co-incubation with aphidicolin ($r_s = 0.453$, $n = 22$, $P < 0.05$). Indeed, when patients were grouped according to in vitro sensitivity to ara-C, the modulation effect of aphidicolin was significantly greater for blasts cells from the resistant group (median 8.9-fold increase in sensitivity compared to 2.12-fold for the sensitive group, $P < 0.01$, Figure 1).

### Correlation between DNA analysis and MTT assay

Figure 2 shows the increase in the subdiploid population of blast cells on co-incubation in ara-C + aphidicolin for 48 hours compared to ara-C alone. There was a significant correlation between the percentage of viable diploid cells as measured by flow cytometry and the percentage cell survival as measured by the MTT assay (Figure 3, $n = 12$, $r = 0.869$). These results validate the use of flow cytometry to study chemosensitivity in these fresh

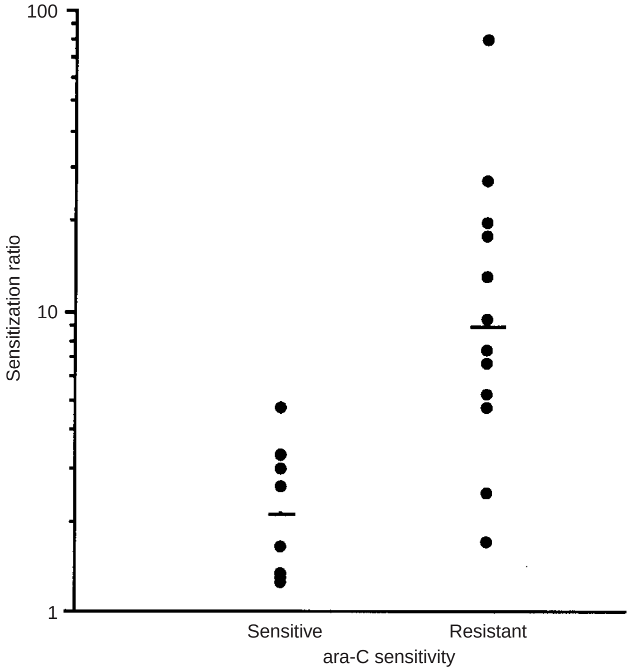

**Figure 1** The effect of aphidicolin according to blast cell sensitivity to ara-C. The sensitization ratio (SR) represents the fold increase in sensitivity found on co-incubation with 15 µM aphidicolin. The median SR (lines) for the resistant group ($n = 15$) was significantly greater than for the sensitive group ($n = 8$; $P < 0.01$)

tumour samples since the same results were achieved as with the MTT assay. The method is simple, reliable and cheap and has the advantage of batching samples for analysis.

### Effect of ara-C ± aphidicolin on cord blood cells

The cells from all but one of the cord blood samples appeared resistant to ara-C with a median $LC_{50}$ value of 8.75 µM (range 1.56–22.6) compared to 5.02 µM (range 0.25–41) for AML blast cells. As with blast cells, sensitivity to ara-C was significantly increased overall after co-incubation with aphidicolin ($P < 0.02$). However, the median sensitization ratio was 3.7 which was not significantly different from that of 2.12 obtained for blasts cells which were sensitive to ara-C. When the therapeutic index of the effect of ara-C on normal cells from cord blood over that for blast cells was calculated (Table 2), the index for ara-C + aphidicolin was higher than that for ara-C alone suggesting this combination may be less myelotoxic than ara-C alone. The median CD34+ stem cell percentage was 2.13% compared to the expected CD34+ population in peripheral blood of 0.01–0.05% (Barnett et al, 1999).

### DISCUSSION

The low overall survival rate in adult AML is normally attributed to the development of drug resistance. The MDR1 phenotype, associated with resistance to the anthracyclines, is important in this disease and numerous attempts have been made to overcome this resistance with MDR modulators but with mixed results (Covelli, 1999). Our results suggest it may be more pertinent to modulate resistance to the commonly used antimetabolite, ara-C. We have shown it is possible to markedly increase the in vitro

© 2001 Cancer Research Campaign

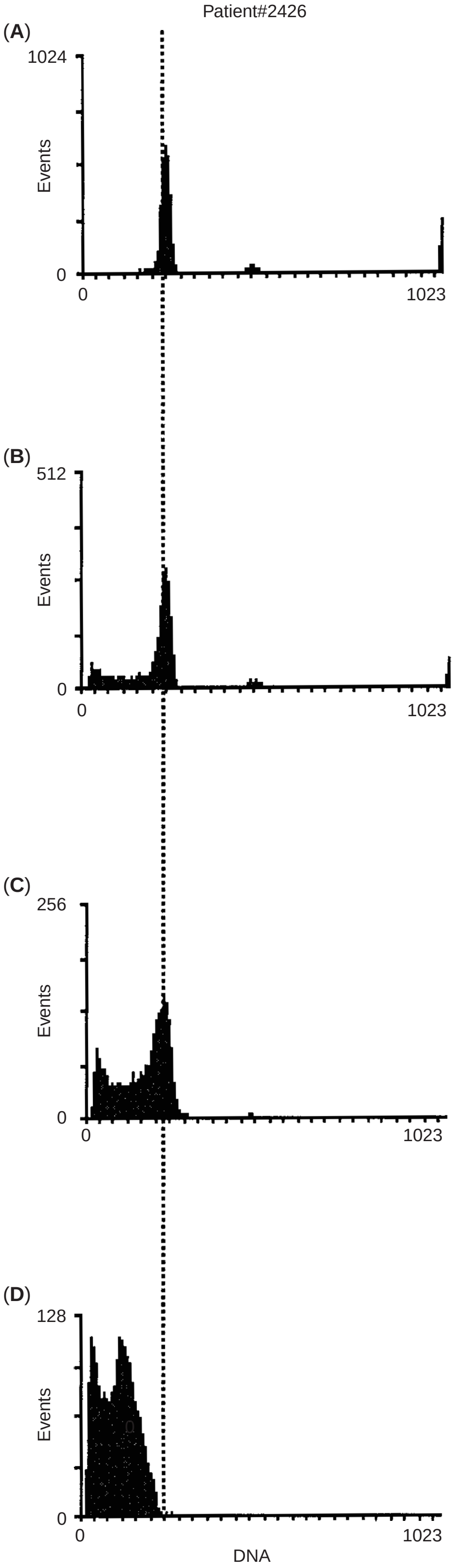

**Figure 2** DNA analysis after 48 hour drug exposure in blast cells from a patient with AML. Cells were fixed then stained with propidium iodide in the presence of RNase. (**A**) culture medium; (**B**) 15 µM aphidicolin; (**C**) 5 µM ara-C; (**D**) the increased effect of 5 µM ara-C + 15 µM aphidicolin is clearly identified in the subdiploid population of cells

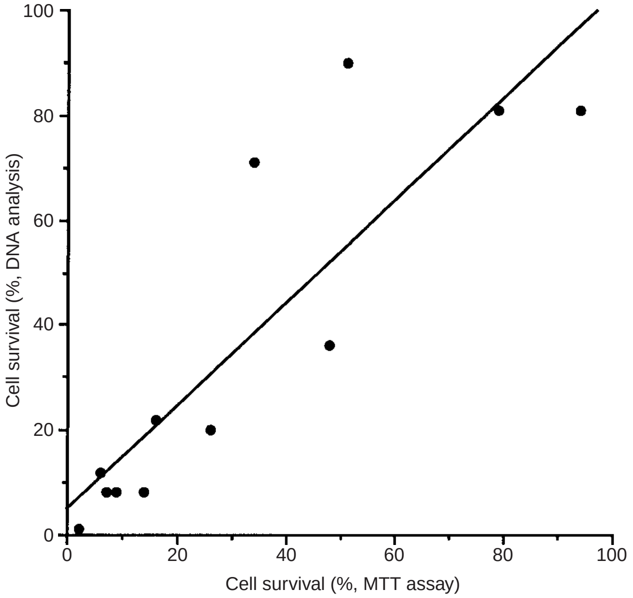

**Figure 3** Relationship between cell survival as measured by the MTT assay and by DNA analysis ($n$ = 12, r = 0.869)

**Table 2** Haematological toxicity of ara-C ± aphidicolin

| | ara-C | ara-C + aph |
|---|---|---|
| Cord blood median $LC_{50}$ (µM) | 8.75 | 1.92 |
| AML blasts median $LC_{50}$ (µM) | 5.02 | 0.91 |
| Therapeutic index Normal cells/tumour cells | 1.74 | 2.1 |

sensitivity to ara-C in these blast cells from clinical samples up to 80-fold using the DNA polymerase inhibitor, aphidicolin.

These results agree with those we found previously, when testing cells from patients with ovarian cancer against the platinum agents in the presence of aphidicolin (Sargent et al, 1996). Here, we found significant increases in sensitivity up to 60-fold. The median increase was 10-fold which is comparable to that found overall for the AML blast cells tested against ara-C (Table 1). Furthermore, over 2-fold increases in sensitivity were seen for the majority of cases in both these tumour types. In the present study, there was only one instance of slightly reduced ara-C cytotoxicity (sensitization ratio = 0.8). This patient appeared to have extreme resistance to ara-C and our results suggest that this was manifested by a mechanism of resistance other than increased DNA repair.

Several studies have shown aphidicolin can increase sensitivity to a variety of drugs, both in cell lines (Kuwakado et al, 1993; Moreland et al, 1999) and in fresh cells obtained from patients with chronic lymphocytic leukaemia (Bramson and Panasci, 1995). Nevertheless, there is one recent report using human lung cancer cell lines where sensitivity to cisplatin was unaffected by aphidicolin, possibly because a 1000-fold lower concentration was used (Heim et al, 2000). We have found that 15 µM aphidicolin is non-cytotoxic in vitro whilst being clinically achievable (Sessa et al, 1991).

© 2001 Cancer Research Campaign

Probably the most interesting and clinically relevant finding of these studies is the increase in the modulation effect of aphidicolin in the group of patients showing in vitro resistance to the drug under test. This observation agrees with the theory that resistant cells often have an increased capacity to repair damaged DNA and suggests that the more resistant the patient to the drug in question the better the effect of aphidicolin, thereby benefiting the patient population most requiring rescue. The positive correlation between ara-C $LC_{50}$ values and the increase in sensitivity afforded by aphidicolin agrees with our previous findings for the platinum agents in ovarian cancer. In this earlier investigation, we found that there was no correlation between the modulation effect of aphidcolin and the number of tumour cells in the final cell population. It was interesting to see that a strikingly similar picture emerged with the tumour cell rich samples in the present study (blast cells >80% in all cases). We previously postulated that enhanced DNA repair may be a feature of 'resistant' tumour cells rather than 'resistant' normal cells and the results obtained from testing cells from cord blood support this hypothesis.

Whilst aphidicolin increased sensitivity to ara-C in the cord blood cells, the median fold-increase was lower than that found for the blast cell population overall, 3.7-fold compared to 4.75-fold. If this comparison is made with the blast cells found resistant to ara-C in vitro (8.9-fold) then the difference becomes more apparent. These data therefore suggest a good therapeutic index for this combination. As only a small percentage of cells in the cord blood samples were stem cells, our results could be misleading. Yet, no difference in the metabolism of ara-C has been found between $CD34^+$ stem cells isolated by FACS and normal bone marrow mononuclear cells (Braess et al, 1999). Because mononuclear cells isolated from cord blood closely resemble the morphology of those from normal bone marrow they can provide a good surrogate for investigations of haematological toxicity (Ghielmini et al, 1997), particularly as they are far easier to obtain.

DNA damage repair can take many forms (Barret and Hill, 1998) usually ending with a re-synthesis step requiring DNA polymerase (pol). The repair mechanism most affected by inhibiting these polymerases with aphidicolin remains the subject of conjecture. Aphidicolin appears to inhibit pol-α, -δ and -ε therefore base excision repair is an unlikely candidate, as pol-β has been described as the major enzyme involved (Wood, 1996). A more likely contender is nucleotide excision repair (NER) as both pol-δ and -ε are known to be responsible for NER synthesis. It would be interesting to investigate the expression of the proteins involved in NER such as XPA and ERCC1/XPF in these fresh blast cells to further elucidate the mechanism responsible for this effect. Mismatch repair (MMR) is also possible, as resynthesis after the mismatch is removed is thought to be carried out by pol-δ (Longley et al, 1997). However, this possibility is confounded by the fact that aphidicolin appears to modulate resistance to cisplatin in MMR defective cells (Moreland et al, 1999).

Despite inhibitors of topoisomerase ll being associated with DNA repair (McKenna and Padua, 1997), we did not see any significant effect of aphidicolin on sensitivity to doxorubicin, daunorubicin or etoposide. Because damage by these agents involves changes in the topology of DNA, increasing cytotoxicity by inhibiting DNA polymerases may not be as pertinent as it is for antimetabolites and alkylating agents, which after incorporation into DNA have to be removed and replaced to repair the damage. Indeed, the involvement of doxorubicin in NER, for example, is still a matter of debate (Barret and Hill, 1998). Also, it could be that modulation of the anthracyclines was low in this study since very few blast cells appeared to be resistant to these agents. It would be interesting to test more samples from previously treated patients on relapse. We are unable to explain the lack of efficacy seen for combining fludarabine with aphidicolin.

Both aphidicolin and ara-C have been described as DNA polymerase inhibitors so questioning the mode of action of this synergistic effect. There is clear evidence to suggest that ara-C may inhibit polymerases indirectly by termination of DNA chain elongation. Once incorporated at the 3′-end of DNA ara-CMP serves as a poor substrate for the addition of a subsequent deoxynucleotide (Plunkett and Ghandi, 1993). Aphidicolin, on the other hand, directly competitively inhibits the enzymes themselves. As a major mechanism of ara-C resistance is conferred by alterations in the enzymes that influence the conversion of ara-C to ara-CTP, it is possible that aphidicolin modulates the rate of intracellular accumulation of ara-CTP. However, studies on proliferating human fibroblasts suggest that this is not the case (Mirzayans et al, 1994). Another mechanism of action for the synergy between ara-C and aphidicolin has been postulated by Kuwakado et al (1995). They found that aphidicolin significantly augmented ara-C-induced c-*jun* upregulation and NF-κB activation in a human myeloid leukaemia cell line. This correlated well with the potentiation by aphidicolin of ara-C-induced apoptosis. As several investigators have described the induction of transcription factors such as c-*jun* and NF-κB as necessary for the apoptotic process, any increase should lead to greater cell kill. Further mechanistic studies are required to elucidate the biochemical or biological basis of the observed modulation of ara-C sensitivity by aphidicolin.

This is the first detailed report of the positive modulation effect of aphidicolin on resistance to ara-C in fresh blast cells from patients with AML. Resistance to ara-C has previously been described as an indicator of reduced disease free survival in this disease and we have found that blast cells from 50–60% of AML patients appear to be resistant in vitro to this agent. Despite these observations ara-C is included in most AML drug regimens, therefore attempts to overcome resistance to this agent become essential. Aphidicolin has already been through clinical trial with minimum toxicity but it lacked significant antitumour activity as a single agent. As has been the fate of other such compounds (Von Hoff, 1998), this promising addition to the cytotoxic repertoire has been shelved. Our study clearly demonstrates the remarkable ability of aphidicolin to modulate resistance to ara-C. This, alongside our increasing understanding of the importance of DNA repair in failure of chemotherapy (Barret and Hill, 1998) make it the ideal candidate for inclusion in future therapeutic programmes for cancer chemotherapy.

### ACKNOWLEDGEMENTS

We would like to thank Philip Bamford, Sheila Walsh and the staff of the Maternity Unit, Pembury Hospital for collecting the cord blood samples. This study was supported by the Tracy Sollis Leukaemia Trust, the EB Hutchinson Trust and the Haematology Research Fund, Pembury Hospital.

### REFERENCES

Åström M, Bodin L, Nilsson I and Tidefelt U (2000) Treatment, long-term outcome and prognostic variables in 214 unselected AML patients in Sweden. *Br J Cancer* **82**: 1387–1392, doi: 10.1054/bjoc.1999.1123

 © 2001 Cancer Research Campaign

Barnett D, Janossy G, Lubenko A, Matutes E, Newland A and Reilly JT (1999) Guideline for the flow cytometric enumeration of $CD34^+$ haematopoietic stem cells. *Clin Lab Haem* **21**: 301–308

Barret J-M and Hill BT (1998) DNA repair mechanisms associated with cellular resistance to antitumor drugs: potential novel targets. *Anti-cancer Drugs* **9**: 105–123

Braess J, Wegendt C, Feuring-Buske M, Riggert J, Kern W, Hiddeman W and Schleyer E (1999) Leukaemic blasts differ from normal bone marrow mononuclear cells and $CD34^+$ haemopoietic stem cells in their metabolism of cytosine arabinoside. *Br J Haematol* **105**: 388–393

Bramson J and Panasci L (1995) Potentiation of chlorambucil toxicity in B-CLL lymphocytes using the DNA synthesis inhibitors aphidicolin and 1-β-D-arabinofuranosylcytosine. *Biochem Pharmacol* **50**: 131–135

Covelli A (1999) Modulation of multidrug resistance (MDR) in hematological malignancies. *Ann Oncol* **10** (Suppl 6): 53–59

Elgie AW, Sargent JM, Alton P, Peters GJ, Noordhuis P, Williamson CJ and Taylor CG (1998) Modulation of resistance to ara-C by bryostatin in fresh blast cells from patients with AML. *Leuk Res* **22**: 373–378

Freund A, Boos J, Harkin S, Schultze-Mosgau M, Veerman G, Peters GJ and Gescher A (1998) Augmentation of 1-β-D-Arabinofuranosylcytosine (Ara-C) cytotoxicity in leukaemia cells by co-administration with antisignalling drugs. *Eur J Cancer* **34**: 895–901

Ghielmini M, Bosshard G, Capolongo L, Geroni MC, Pesenti E, Torri V, D'lncalci M, Cavalli F and Sessa C (1997) Estimation of the haematological toxicity of minor groove alkylators using tests on human cord blood cells. *Br J Cancer* **75**: 878–883

Heim MM, Eberhardt W, Seeber S and Muller MR (2000) Differential modulation of chemosensitivity to alkylating agents and platinum compounds by DNA repair modulators in human lung cancer cell lines. *J Cancer Res Clin Oncol* **126**: 198–204

Klumper E, Ossenkoppele GJ, Pieters R, Huismans DR, Loonen AH, Rottier A, Westra G and Veerman AJP (1996) *In vitro* resistance to cytosine arabinoside, not to daunorubicin, is associated with the risk of relapse in *de novo* acute myeloid leukaemia. *Br J Haematol* **93**: 903–910

Kuwakado K, Kubota M, Hirota H, Adachi S, Matsubara K, Kasai Y, Akiyama Y and Mikawa H (1993) Aphidicolin potentiates apoptosis induced by arabinosy9 nucleosides in human myeloid leukemia cell lines. *Biochem Pharmacol* **46**: 1909–1916

Kuwakado K, Kubota M, Bessho R, Kataoka A, Usami I, Wei Lin Y, Okuda A and Wakazono Y (1995) Augmentation by aphidicolin of 1-β-D-arabinofuranosylcytosine-induced c-*jun* and NF-κB activation in a human myeloid leukemia cell line: correlation with apoptosis. *Leuk Res* **19**: 645–650

Longley MJ, Pierce AJ and Modrich P (1997) DNA polymerase δ is required for human mismatch repair *in vitro. J Biol Chem* **272**: 10917–10921

McKenna SL and Padua RA (1997) Multidrug resistance in leukaemia. *Br J Haematol* **96**: 659–674

Mirzayans R, Enns L, Cubitt S, Karimian K, Radatus B and Paterson MC (1994) Effect of DNA polymerases inhibitors on repair of γ ray-induced DNA damage in proliferating (intact versus permeable) human fibroblasts: evidence for differences in the modes of action of aphidicolin and 1-β-D-arabinofuranosylcytosine. *Biochim Biophys Acta* **1227**: 92–100

Moreland NJ, Illand M, Kim YT, Paul J and Brown R (1999) Modulation of drug resistance mediated by loss of mismatch repair by the DNA polymerase inhibitor aphidicolin. *Cancer Res* **59**: 2102–2106

Plunkett W and Ghandi V (1993) Cellular pharmacodynamics of anticancer drugs. *Sem Oncol* **20**: 50–63

Sargent JM and Taylor CG (1989) Appraisal of the MTT assay as a rapid test of chemosensitivity in acute myeloid leukaemia. *Br J Cancer* **60**: 206–210

Sargent JM, Elgie AW, Williamson CJ and Taylor CG (1996) Aphidicolin markedly increases the platinum sensitivity of cells from primary ovarian tumours. *Br J Cancer* **74**: 1730–1733

Sargent J, Elgie A, Williamson C and Taylor C (1997) The use of the MTT assay to study drug resistance in acute myeloid leukaemia. *Adv Blood Dis* **3**: 33–41

Sargent JM, Elgie AW, Williamson CJ and Taylor CG (1999) Aphidicolin markedly increases the *in vitro* sensitivity to ara-C of blast cells from patients with AML. *Adv Exp Med Biol* **457**: 567–570

Sessa C, Zucchetti M, Davoli E, Califano R, Cavalli F, Fristaci S, Gumbrell L, Sulkes A, Winograd B and D'Incalci M (1991) Phase I and clinical evaluation of aphidicolin glycinate. *J Natl Cancer Inst* **83**: 1160–1164

Sherwood SW and Schimke RT (1995) Cell cycle analysis of apoptosis using flow cytometry. *Methods Cell Biol* **46**: 77–97

Von Hoff DD (1998) There are no bad anticancer agents, only bad clinical trial designs – Twenty-first Richard and Hilda Rosenthal Foundation Award Lecture. *Clin Cancer Res* **4**: 1079–1086

Wood RD (1996) DNA repair in eukaryotes. *Annu Rev Biochem* **65**: 135–167

*© 2001 Cancer Research Campaign*
